# Potential implications of subclinical hypervolemia, identified by a multiparametric approach, in causing left ventricular hypertrophy in clinically euvolemic children on dialysis: a prospective longitudinal pilot study

**DOI:** 10.1007/s00467-026-07159-z

**Published:** 2026-01-30

**Authors:** Marco Allinovi, Valentina Querin, Silvia Menale, Andrea La Tessa, Alice Brambilla, Ilaria Farella, Luigi Cirillo, Gianmarco Lugli, Carmela Errichiello, Giulio Porcedda, Francesca Miselli, Martina Giacalone, Francesca Becherucci

**Affiliations:** 1 Nephrology, Dialysis and Transplantation Unit, Careggi University Hospital, Florence, Italy; 2 Division of General Cardiology, Cardiothoracovascular Department, Azienda Ospedaliero-Universitaria Careggi and University of Florence, Florence, Italy; 3 Nephrology and Dialysis Unit, Meyer Children’s Hospital IRCCS, Florence, Italy; 4 Department of Emergency Medicine and Trauma Center, Meyer Children’s Hospital IRCCS, Florence, Italy; 5 Department of Precision and Regenerative Medicine and Ionian Area, Clinica Medica “A. Murri”, University of Bari “Aldo Moro”, Bari, Italy; 6 Pediatric Cardiology Unit, Meyer Children’s Hospital IRCCS, Florence, Italy; 7 PhD Program in Clinical and Experimental Medicine, University of Modena and Reggio Emilia, Modena and Reggio Emilia, Italy; 8 Department of Biomedical, Experimental and Clinical Sciences “Mario Serio”, University of Florence, Florence, Italy

**Keywords:** Chronic dialysis, Subclinical hypervolemia, Left ventricular hypertrophy, Fluid overload

## Abstract

**Background:**

Fluid overload in children undergoing dialysis can lead to serious cardiac complications, i.e., left ventricular hypertrophy (LVH) and cardiac dysfunction. Studies investigating the cardiovascular effects of persistent subclinical hypervolemia—characterized by euvolemia at clinical assessment but hypervolemia at technical evaluation—are lacking. This pilot study explored the combined use of lung ultrasound (LUS), bioimpedance spectroscopy (BIS), and ultrasound assessment of the inferior vena cava collapsibility index (IVC-CI) to identify subclinical hypervolemia and investigated its cardiac impact.

**Methods:**

In this longitudinal study, we recruited 23 children on chronic dialysis who underwent fluid status evaluation (physical examination, LUS, IVC-CI, BIS) every 2 months and echocardiography every 6 months.

**Results:**

In clinically euvolemic patients, we observed a significant positive correlation between the interdialytic weight gain and the number of B-lines observed by LUS (*R* = 0.2923, *p* < 0.001); similar results were obtained for the OH/ECW measured by BIS (*R* = 0.4144, *p* < 0.001), while a negative correlation with IVC-CI (*R* =  − 0.2597, *p* = 0.019) was observed. Moreover, we identified a significant linear correlation between left ventricular mass index values and the average pre-dialysis systolic blood pressure measured over the preceding 6 months (*R*^2^ = 0.16, *p* = 0.002). Hospitalizations due to hypertensive crises (67% vs. 0%, *p* < 0.01) and the occurrence of LVH at the final follow-up (75% vs. 27%, *p* = 0.04) were notably more frequent in children with subclinical hypervolemia.

**Conclusion:**

In clinically euvolemic children on dialysis, the combined use of LUS, BIS, and IVC-CI (multiparametric approach) effectively quantified subclinical hypervolemia, which was correlated with the risk of LVH.

**Graphical abstract:**

**Figure:**
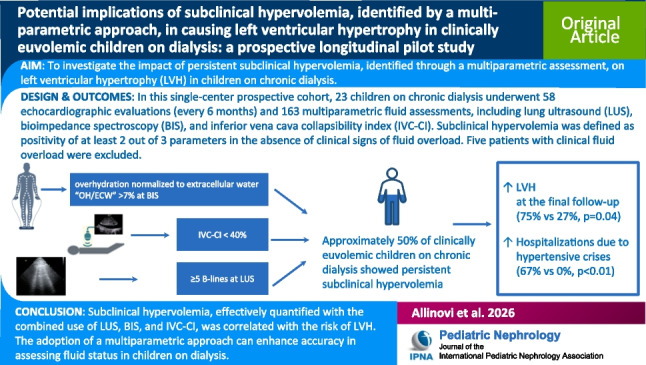
A higher resolution version of the Graphical abstract is available as Supplementary information

**Supplementary Information:**

The online version contains supplementary material available at 10.1007/s00467-026-07159-z.

## Background

Hypervolemia is a common problem in pediatric patients undergoing chronic dialysis, potentially leading to adverse outcomes, including left ventricular hypertrophy (LVH) and cardiac dysfunction. In the pediatric dialysis population, the prevalence of LVH varies between 60 and 85% [1–4]. Therefore, early interventions aimed to normalize blood pressure (BP) and fluid balance are vital, tailoring treatments and preventing progression to heart failure [5]. Despite these observations, an accurate assessment of fluid status is particularly difficult in growing children on dialysis. The dry weight, defined as the lowest tolerated post-dialysis weight associated with minimal signs or symptoms of fluid overload, is largely determined empirically and changes frequently. Indeed, linear growth, weight loss due to concurrent diseases, and many additional clinical reasons make the estimation of dry weight in the pediatric dialysis population particularly challenging. Consequently, children are exposed to a high risk of intradialytic hypotension, which has been shown to be associated with acute and long-term adverse events and mortality [6, 7]. In addition, studies performed in the adult population have shown that the decline in systolic pressure is an important risk factor for myocardial stunning and cerebral ischemia, with a consequent increased risk of developing dementia [8, 9].

Even if several non‐invasive methods for assessing fluid balance are emerging, each measurement taken separately has different strengths and diagnostic limitations in sensitivity, specificity, and predictive value [10]. Indeed, bedside evaluation is often inadequate, as various factors other than hydration can affect clinical parameters and compromise their accuracy [11]. Therefore, determining a patient’s fluid status prior to dialysis treatment remains challenging and may require a combination of clinical parameters and technical measurements (e.g., lung ultrasound (LUS), bioimpedance spectroscopy (BIS), chest X-ray, or natriuretic peptides).

While clinically quantified hypervolemia in adults has widely demonstrated a prognostic association with various strong outcomes (including death, cardiovascular events, decompensated heart failure), the role of subclinical hypervolemia, defined as a status of euvolemia at clinical assessment but hypervolemia at technical evaluation, is far from being defined. Only a few studies have adopted different techniques for fluid status assessment simultaneously in children, with inconclusive results regarding which method or combination of methods is the most sensitive and specific in predicting subclinical hypervolemia [12, 13].

Furthermore, most pediatric dialysis patients with moderate hypervolemia (at LUS or BIS) appear clinically euvolemic and with normal blood pressure [14], and no studies in this population have evaluated the potential cardiovascular impact of prolonged subclinical hypervolemia. Recent studies in adult patients on dialysis showed that even a moderate pre-dialysis fluid overload correlates with overall survival and/or cardiovascular morbidity [15].

The aim of this pilot study was to evaluate the combined use of the three most studied methods for detecting subclinical hypervolemia in children on dialysis: B-line quantification by LUS [16, 17], BIS by Body Composition Monitor (BCM) Fresenius [18–20], and ultrasound assessment of inferior vena cava collapsibility index (IVC-CI) [21, 22]. Considering that every method has some limitations, we also prospectively tested a simultaneous combination of available measurements of different methods for predicting hypervolemia. Finally, we evaluated the potential implications of persistent subclinical hypervolemia in causing LVH.

## Methods

### Study population

In this prospective, longitudinal, monocentric, observational pilot study, we evaluated and recruited all consecutive children (both prevalent and incident) on chronic hemodialysis or peritoneal dialysis in the regional pediatric nephrology center of Meyer Children’s Hospital IRCSS, Florence, Italy, between 01/06/2018 and 01/11/2020.

Inclusion criteria were (1) available information on the serial multiparametric evaluations of their fluid status and echocardiography, (2) at least 12 months of follow-up, and (3) age < 18 years.

Exclusion criteria were (1) clinical fluid overload, defined as an excess total body water content associated with physical signs/symptoms consistent with volume excess, including weight gain, edema, crackles, increased skin turgor, and/or elevated blood pressure; (2) co-existent pulmonary or cardiac pathologies (such as interstitial lung disease, heart failure, or congenital heart defects).

The study was conducted in accordance with the Declaration of Helsinki. The local Ethics Committee “Comitato Etico Regionale per la Sperimentazione Clinica della Regione Toscana” approved the study protocol (study approval number SFOS2018). Parents or legal representatives gave full written informed consent.

### Multiparametric approach of status assessment

For each patient, we performed a multiparametric evaluation of fluid overload, including clinical assessment, LUS and abdominal US for IVC-CI, and BIS. Multiparametric evaluation was performed bimonthly in the mid-week dialysis session.

**Fig. 1:**
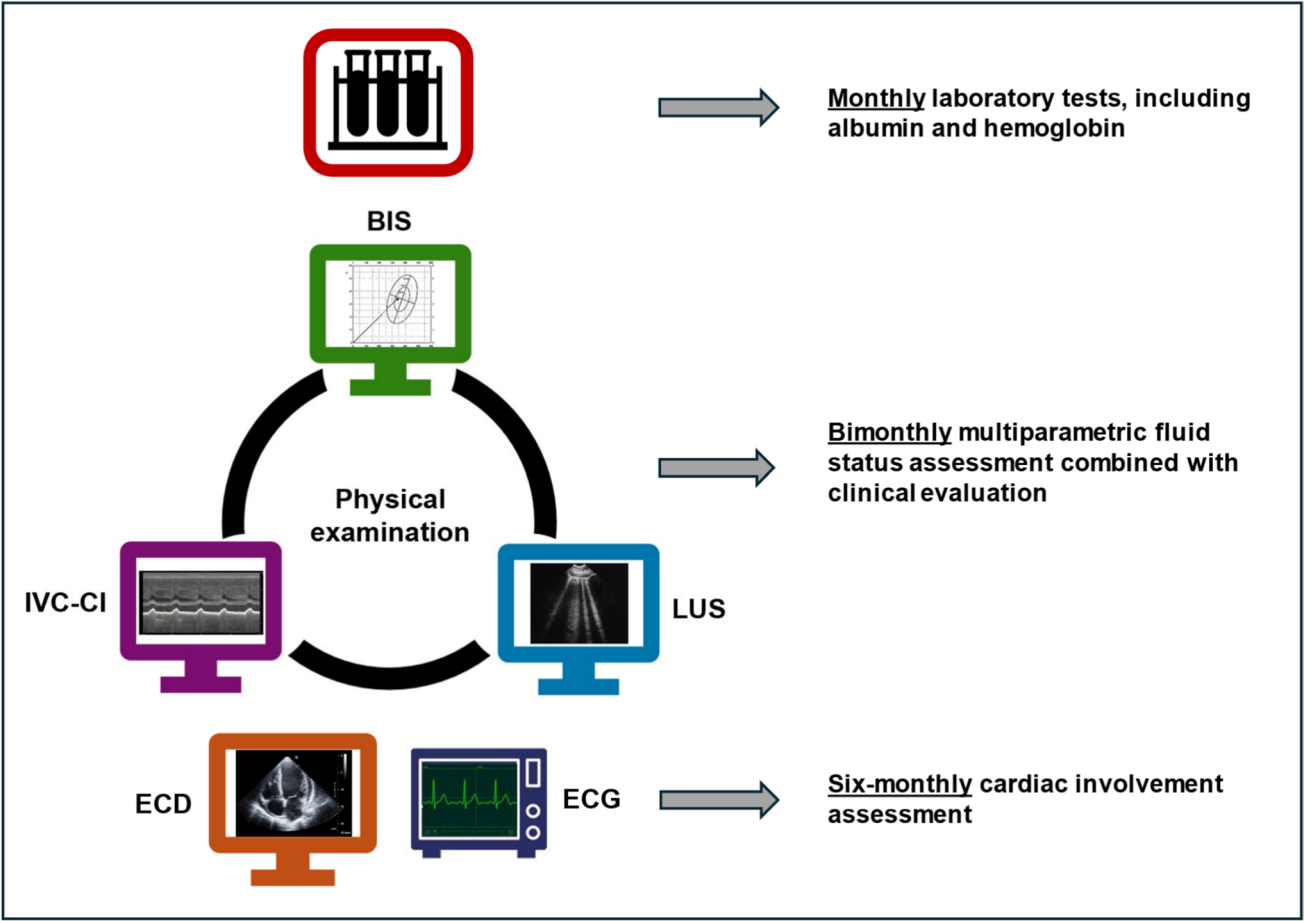
Clinical and instrumental investigations performed on patients during the study. *BIS* bioimpedance spectroscopy, *LUS* lung ultrasound, *IVC-CI* inferior vena cava collapsibility index, *ECD* echocardiography, *ECG* electrocardiogram

Each patient underwent multiple multiparametric evaluations of fluid status and echocardiography at different time-points (Fig. 1), for at least 12 months of follow-up.

### Clinical evaluation

The overload rate by weight (weight-derived OH) was expressed as the proportional increase from dry weight (kg of fluid overload/dry weight in kg × 100), also called the percentage of body weight gain. The dry weight was that prescribed by the physician in charge of the patient using clinical data, including physical examination (weight gain, crackles, skin turgor, and edema), BP, and intradialytic symptoms.

The BP was measured using a standard mercury sphygmomanometer. Because of the fluctuating nature of pre-dialysis BP, predialytic systolic BP and diastolic BP were recorded from consecutive hemodialysis sessions during a 1-month (12 routine measurements) period. BP measurement was conducted by three readings on the non-arterio-venous fistula. We used the last BP value of the pre-dialysis recording. We defined systemic hypertension as systolic BP and/or diastolic BP ≥ 95th percentile for age and height for both boys and girls, or over 120/80 mmHg [23]. Resistant hypertension was defined as elevated BP above target in clinically euvolemic patients despite concurrent use of three antihypertensive drugs from different drug classes. Ambulatory blood pressure monitoring (ABPM) was not systematically performed, and therefore BP classification relied on averaged pre-dialysis measurements.

### Lung ultrasound

Total number of lung B-lines was assessed and counted in 28 intercostal positions over the antero-lateral chest, as previously reported [14, 17]. For patients with a body weight below 20 kg, we used a 14-position assessment (parasternal, midclavicular, and anterior axillary/midaxillary sites). Euvolemia was defined as < 5 B-lines, while B-lines ≥ 5 were considered indicative of lung congestion and hypervolemia [24], regardless of the presence of clinical signs/symptoms of fluid overload.

### Ultrasound of the inferior vena cava

Inferior vena cava diameters (IVCD) were measured by abdominal US from subxiphoid long axis position in 2 cm to its junction to right atrium, in supine position. The maximum diameter in expiration (MDe) and the minimum diameter in deep inspiration (MDi) were measured and indexed for body surface area (BSA). IVC-CI was determined by the percentage of the ratio (MDe − MDi)/MDe. Normal values of the IVC-CI were set between 75 and 40%. Values greater than 75% were considered a sign of dehydration, whereas values < 40% were indicative of hypervolemia [25], regardless of the presence of clinical signs/symptoms.

### Bioimpedance spectroscopy

The BCM (Fresenius Medical Care, Germany) was employed to measure the BIS at 50 frequencies (5 kHz to 1000 MHz), enabling the estimation of total body water, extracellular water, and intracellular water. A normal range of fluid status was defined as − 7% to + 7%, corresponding to the 10th and 90th percentiles of a reference healthy population (whose data is saved in the device). Accordingly, a Relative Tissue Hydration (OH/ECW % = hydration normalized to extracellular water) greater than 7% was classified as “hypervolemia” [26, 27], regardless of the presence of clinical signs/symptoms of fluid overload.

### Definition of subclinical hypervolemia and identification of separate groups

After excluding children with clinically evident fluid overload, patients were divided in two groups depending on fluid status detected by the three validated different methods: B-line quantification by LUS, BIS, and assessment of IVC-CI.

An average value of each fluid status measurement with different methods (B-lines, BIS, IVC-CI) detected during the entire study was performed, identifying an “average B-line number”, “average OH/ECW”, and “average IVC-CI”. In relation to these values, a chronic subclinical hypervolemia (group SH) was defined in a patient with average values of fluid overload (average B-line number ≥ 5, average OH/ECW > 7%, average IVC-CI < 40%) in at least two out of three methods (LUS, BIS, and/or IVC-CI) in the absence of clinical signs/symptoms of hypervolemia. Euvolemia (group EU) was an exclusion diagnosis.

### Echocardiography

Evaluation of cardiac involvement included 12-lead ECGs and transthoracic two-dimensional, color Doppler, tissue Doppler, and M-mode echocardiography, interpreted according to standard criteria. Echocardiography was performed every 6 months on a non-dialysis day (days between, not the longest day), preferably after the midweek hemodialysis session, in order to avoid the effect of volume changes on LV mass. Measurements were indexed to age and BSA, and corresponding *z*-scores were derived. LVH was defined by a left ventricular mass index (LVMI) ≥ 95th percentile using age- and body-surface adjusted reference values [26, 28]. This percentile-based definition is the standard approach in pediatric CKD and dialysis studies and differs from the fixed LVMI cutoff proposed for older children in the pediatric hypertension guideline. LV wall thickness > 2 *z*-scores was also required for infants (1 month – 1 year), children (1–12 years), and adolescents (13–17 years) [29]. Dilated cardiomyopathy was defined by increased ventricular end-diastolic diameter (> 2 *z*-scores) with systolic dysfunction. Left ventricular function was assessed on the basis of ejection fraction (LVEF) and systolic index of contractility (dP/dt, normal value > 1200 mmHg/sec, severe reduction if < 800 mmHg/s). FS was measured in M-mode, and LVEF was measured by the biplane Simpson method (normal value > 50%, mild dysfunction 40–49%, moderate dysfunction 30–39%, severe dysfunction < 30%). Diastolic dysfunction was declared in case of abnormal mitral E/A ratio and deceleration time and increased E/e′ ratio [30].

### Data collection

Physicians performing clinical examinations and multiparametric fluid status assessment (LUS, BIS, IVC-CI) and those performing echocardiography were aware of the respective results and of the patients’ dry weight.

For each patient we collected the following clinical data: age, sex, BSA, type of dialysis treatment, and residual diuresis. Oliguria was defined as urine output < 0.5 mL/kg/h for 24 h in infants or < 500 mL/1.73 m^2^/day in older children. In addition, we recorded monthly measurements of hemoglobin and serum albumin for each patient from enrollment (Fig. 1).

### Statistical analysis

Parametric data are presented as mean ± standard deviation, while nonparametric data are reported as median and interquartile range (from 25 to 75th percentile). Normality was assessed using the Shapiro–Wilk test. Normally distributed continuous variables were compared using Student’s *t* test, whereas the non-parametric Mann–Whitney *U* test was used for other variables. Proportions were compared using the Chi-square test. Correlations between variables were assessed using either the Pearson product-moment correlation coefficient or the Spearman rank correlation coefficient, as appropriate. Simple univariable linear regression models were used to quantify the relationship between weight-derived OH and each instrumental parameter (B-lines, OH/ECW, and IVC-CI). No multivariable regression analyses were performed due to the limited sample size and the risk of overfitting. A *p* value < 0.05 was considered statistically significant. Statistical analyses were performed using SPSS version 22.0 (IBM, Armonk, NY, USA).

## Results

### Patient characteristics

After the application of inclusion and exclusion criteria, 23 children undergoing chronic dialysis treatment (13 hemodialysis, ten peritoneal dialysis) were enrolled in the study (Table 1). Five patients were not included in the final analysis due to the frequent presence of signs/symptoms of fluid overload. In total, the recruited patients underwent 58 bi-annual echocardiography evaluations (every 6 months) and 163 multiparametric fluid assessments. We performed a mean of 7.1 multiparametric evaluations in each patient. Eleven patients had 18 months of follow-up, while 12 patients reached a 12-month follow-up. According to the results of multiparametric evaluations, patients were divided into two groups (see Methods for details): subclinical hypervolemia (SH) and euvolemia (EU). The two groups did not differ with regard to gender, age, and BSA (Table 1).

**Table 1:** Characteristics of the studied cohort of children on chronic dialysis. Data for categorical variables are presented as numbers and percentages; data for continuous variables, as mean ± standard deviation. *p* values refer to comparisons between the subclinical hypervolemia (SH) and euvolemia (EU) groups

	Overall cohort *n* = 23	Subclinical hypervolemia *n* = 12	Euvolemia *n* = 11	*p* value
Male gender, *n* (%)	13 (57)	8 (67)	5 (45)	0.41
Age, years	10 ± 5.5	9.4 ± 6.50	9.8 ± 4.8	0.63
BSA, sqm	1.00 ± 0.47	1.04 ± 0.57	0.97 ± 0.36	0.96
BMI	− 0.39 ± 1.77	− 0.11 ± 1.96	− 0.50 ± 1.64	0.7972
weight-derived OH, %	1.80 ± 2.01	2.20 ± 1.84	1.30 ± 2.16	** < 0.01**
Hemodialysis (versus peritoneal dialysis), *n* (%)	13 (57)	7 (58)	6 (55)	0.81
Oligoanuria, *n* (%)	8 (35)	7 (58)	1 (9)	**0.03**
Hypertension, *n* (%)	18 (78)	10 (83)	8 (73)	0.64
Treatment-resistant hypertension, *n* (%)	3 (13)	3 (25)	0 (0)	0.11
Hospital admissions for fluid-related hypertensive crises, *n* (%)	8 (35)	8 (67)	0 (0)	** < 0.01**
Hospital admissions for fluid-unrelated hypertensive crises, *n* (%)	7 (30)	3 (25)	4 (36)	0.67
Hemoglobin, g/dL (n.v. 11.5–15.5)	10.7 ± 1.53	10.59 ± 1.56	10.77 ± 1.52	0.97
Serum albumin, g/dL (n.v. 3.8–5.4)	3.68 ± 0.52	3.50 ± 0.57	3.86 ± 0.38	**0.02**
Average OH/ECW% > 7%, *n* (%)	11 (48)	10 (83)	1 (9)	** < 0.01**
Average B-line number > 5, *n* (%)	10 (43)	9 (75)	1 (9)	** < 0.01**
Average IVC-CI < 40%, *n* (%)	12 (52)	8 (67)	4 (36)	0.22
LVH at last follow-up, *n* (%)	12 (52)	9 (75)	3 (27)	**0.04**
LVH regression in the last 12–18 months, *n* (%)	3 (13)	1 (8)	2 (18)	0.59
LVH development in the last 12–18 months, *n* (%)	3 (13)	3 (25)	0 (0)	0.11
Diastolic dysfunction at last follow-up, *n* (%)	6 (26)	5 (42)	1 (9)	0.16

*BIS*, bioimpedance spectroscopy; *BSA*, body surface area; *OH*, overhydration; *OH/ECW*, overhydration normalized to extracellular water; *IVC-CI*, inferior vena cava collapsibility index; *LVH*, left ventricular hypertrophy; *weight-derived OH*, also called the percentage of body weight gain, was obtained by the formula: kg of fluid overload/dry weight in kg × 100

The underlying causes of kidney failure in the study cohort included congenital anomalies of the kidney and urinary tract (CAKUT, *n* = 8), glomerular diseases (*n* = 6), genetic diseases (*n* = 7), and other or unknown etiologies (*n* = 2).

In our cohort of apparently euvolemic children on dialysis, about 50% of them showed subclinical hypervolemia when detected with different instrumental methods.

By evaluating the hemodialysis and peritoneal dialysis population it emerged that they should not be compared for their echocardiographic and fluid status parameters because they are significantly different in terms of age (11.6 vs. 7 years), BSA (1.2 vs. 0.7 square meters), and dialysis vintage (22.4 vs. 45.6 months).

### Patients with subclinical hypervolemia appear as a separate group from patients with euvolemia

The weight-derived OH (2.2 ± 1.84 vs. 1.30 ± 2.16%, *p* < 0.01) and the prevalence of oligoanuria (58% vs. 9%, *p* = 0.03) were significantly higher in the SH population than in the EU group. The frequency of hypertension did not differ between the two groups (83% vs. 73%, *p* = 0.64). Of note, treatment-resistant hypertension was detected only in the SH group (25% vs. 0%, *p* = 0.11). Twelve patients experienced at least one hospital admission for hypertensive crises; they all belonged to the SH group (67% vs. 0%, *p* < 0.01). Among laboratory results, serum albumin was significantly lower in the SH group (35.0 ± 5.7 g/dl vs. 38.6 ± 3.8 g/dl, *p* = 0.02), while hemoglobin levels were comparable in the two groups (*p* = 0.97) (Table 1).

Among the three different methods that were adopted in the study for the detection of a state of subclinical hypervolemia, an average OH/ECW > 7% at BIS (83% vs. 9%, *p* < 0.01) and an average B-line number > 5 at LUS (75% vs. 9%, *p* < 0.01) were significantly more frequent in the SH group than in the EU group. Conversely, an average IVC-CI < 40% was more frequent in the SH group but without reaching a significance (67% vs. 36%, *p* = 0.22).

### Different methods for quantifying hypervolemia are significantly correlated with weight-derived OH and with each other

**Fig. 2:**
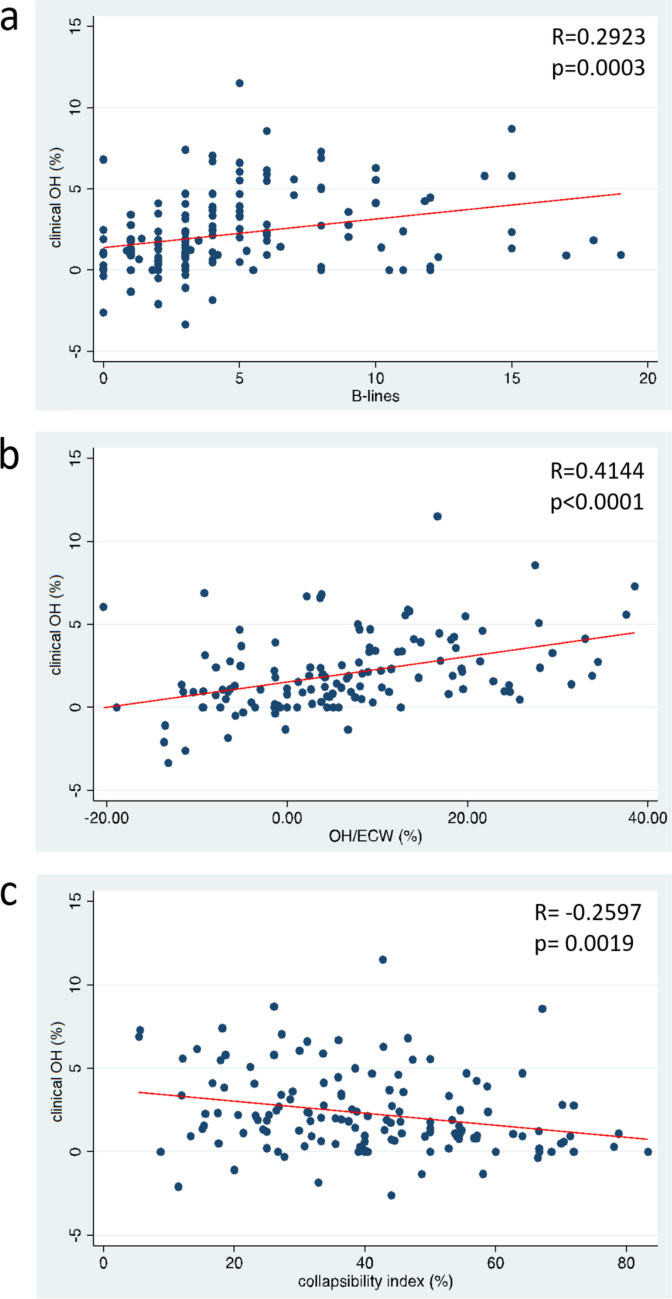
Scatter plots representing the correlation of fluid parameters at different methods with proportional increase in weight from the prescribed target weight. Weight-derived OH (%) significantly correlated with the number of B-lines at LUS (**a**), with OH/ECW% at BIS (**b**), and with IVC-CI (%) (**c**). Legend: Weight-derived OH, proportional (%) increase in weight from the prescribed target weight; OH/ECW, overhydration/extracellular water at bioimpedance spectroscopy (BIS)

We then performed a correlation analysis between the different methods used to quantify subclinical hypervolemia. We built scatter plots taking into account single values of each multiparametric fluid status evaluation (i.e., weight-derived OH, B-lines number, OH/ECW and IVC-CI; Fig. 2). We found that clinical OH had a positive correlation with the number of B-lines (*R* = 0.2923, *p* < 0.001) and OH/ECW (*R* = 0.4144, *p* < 0.001), while we recorded a negative correlation with IVC-CI (*R* =  − 0.2597, *p* = 0.019) (Table 2). The number of B-lines showed a negative correlation with IVC-CI (*R* =  − 0.1699, *p* = 0.0377), while IVC-CI did not significantly correlate with OH/ECW (Table 2). A regression analysis showed weight-derived OH and B-lines significantly correlated (*p* = 0.005), where we expected to see an increase in weight-derived OH of 0.174% for every one-unit increase in B-lines. Similarly, for weight-derived OH and OH/ECW, we expected to see an increase in weight-derived OH of 0.076% for every one-unit increase in OH/ECW (*p* < 0.001). For weight-derived OH and IVC-CI we found an almost significant model (*p* = 0.057) with an opposite regression, where for every one-unit increase in IVC-CI, we expected to see a decrease in weight-derived OH of 0.361% (Table 3).

**Table 2:** Correlation analysis of the methods adopted to detect subclinical hypervolemia

Correlation analysis		
Weight-derived OH		
	Type of correlation (*R* value)	*p*-value
B-lines	0.29	<0.001
OH/ECW	0.41	<0.001
IVC-CI	–0.26	<0.019
B-lines		
OH/ECW	0.34	<0.001
IVC-CI	–0.17	0.0377

**Table 3:** Regression analysis of the methods adopted to detect subclinical hypervolemia

Regression analysis		
Weight-derived OH		
	*R*^*2*^	*p* *value*
B-lines	0.060	**0.0055**
OH/ECW (%)	0.205	** < 0.0001**
IVC-CI (%)	0.031	0.0561
Multiple regression model		
Weight-derived OH		
	*p* *value*	***Model R***^***2***^** = *0.1775***
B-lines	0.9655	
IVC-CI (%)	0.6634	
OH/ECW (%)	**0.0001**	

Although the small sample size limits the strength of these correlations, the consistency across methods supports their potential complementary role in assessing subclinical hypervolemia. In our cohort, LUS, BIS, and IVC-CI failed to predict the correct dry weight in most of the assessments in 3 (13%), 3 (13%), and 6 (26%) patients, respectively.

### Left ventricular mass index correlates with pre-dialysis systolic blood pressure and is higher in children with subclinical hypervolemia

**Fig. 3:**
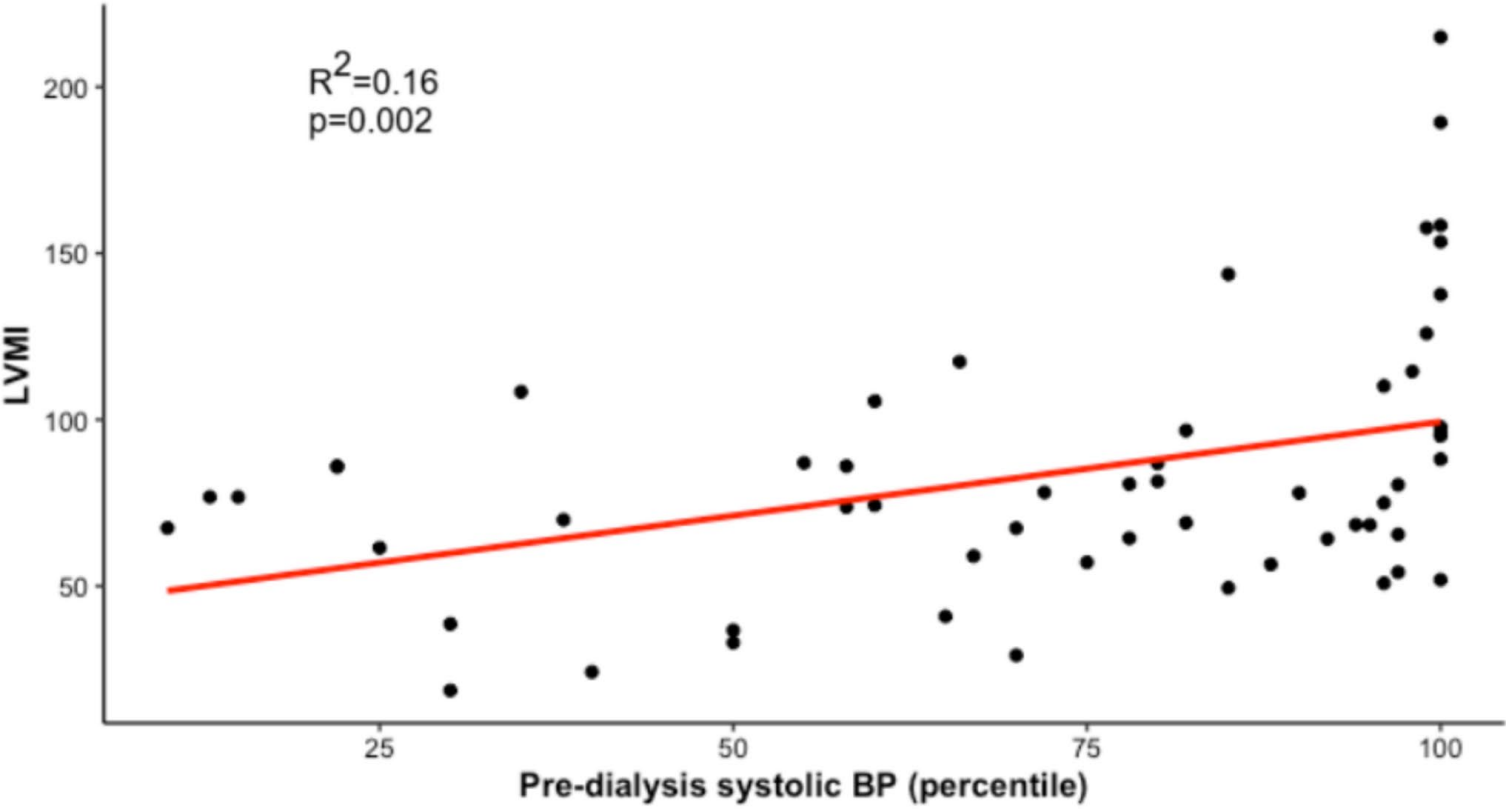
Correlation between LVMI and pre-dialysis systolic blood pressure

A significant linear correlation was found between LVMI values and the average pre-dialysis systolic BP recorded over the preceding 6 months (*r*^2^ = 0.16, *p* = 0.002) across the entire cohort (Fig. 3). Conversely, we did not find a significant correlation between LVMI values and the average values obtained in the prior 6 months of Hb (*p* = 0.85), serum albumin (*p* = 0.1), dialysis vintage (*p* = 0.43), B-lines (*p* = 0.80), IVC-CI (*p* = 0.29), and OH/ECW (*p* = 0.64).

We then compared the results obtained in the SH and EU groups. At the last follow-up, the presence of LVH, measured by the LVMI, was significantly more prevalent in the SH group (75% vs. 27%, *p* = 0.04). Diastolic dysfunction was identified in six patients at last follow-up. Although it was observed more frequently in the SH group (42% vs. 9%), the difference was not statistically significant (*p* = 0.16), while systolic dysfunction was never identified in the study population.

## Discussion

Hypervolemia is generally defined as the extent of a positive fluid balance linked to negative patient outcomes, but the threshold and timing of fluid balance defining a pathologic fluid status differ across populations, depending on the method adopted for quantifying fluid overload [31].

In this pilot study, which included a small cohort of pediatric patients, we found that even an excess of fluids that is not clinically manifest (subclinical hypervolemia)—and which can only be identified and quantified using different techniques—appears to be common in children on dialysis and may be associated with adverse cardiovascular findings. These preliminary observations suggest potential clinical relevance that warrants confirmation in larger, multicenter studies.

We proposed for the first time a potential definition of subclinical hypervolemia, which was supported by its association with LVH development or regression at last follow-up. To the best of our knowledge, our research project represents the first prospective study assessing the combined use of three different methods to detect subclinical hypervolemia in children on dialysis and to explore its possible association with echocardiographic abnormalities. Despite the limited sample size, our findings indicate that subclinical hypervolemia is frequent in children undergoing chronic dialysis and is often underestimated by clinical evaluation alone.

Notably, most pediatric dialysis patients with moderate to severe fluid overload are asymptomatic. Clinical evaluation alone is known to have a very low sensitivity in assessing hypervolemia, potentially underestimating even severe fluid overload.

In our study, after excluding children with overt fluid overload, subclinical hypervolemia was identified in about 50% of children on dialysis who appeared euvolemic by clinical examination when detected with different instrumental methods (group SH). This finding underscores how common subclinical fluid overload can be and highlights the need for sensitive detection methods. As previously demonstrated [14, 27], even in our cohort LUS and BIS appeared to be the most promising and reliable methods to evaluate fluid status and quantify hypervolemia in children on dialysis.

Moreover, although there is no statistical significance, patients with subclinical hypervolemia were more likely to present treatment-resistant hypertension.

In addition, subclinical hypervolemia was more frequently observed in children who had LVH at last follow-up and/or required hospital admissions for fluid-related hypertensive crises. As known, LVH and abnormal LV geometry in children with stage 5 chronic kidney disease (CKD 5) are already present at the time of dialysis initiation, suggesting that LVH may begin prior to kidney failure. The prevalence of LVH in this population ranges from 60 to 85%, and it is commonly found in association with hypertension and volume overload. Very importantly, cardiovascular complications represent the leading cause of death in pediatric patients with kidney failure, accounting for 40–45% of mortality [32]. As a matter of fact, the NKF/KDOQI guidelines for cardiac disease in dialysis patients advise a strict echocardiographic monitoring of children on dialysis after the dry weight is attained, as LVH may worsen or fail to regress in these patients [33]. Cardiovascular changes are not easily reversible, but LVH regression is possible. Early intervention to control and normalize BP and fluid balance (reduction of the extracellular volume, adjusting dry weight) may be an important factor to improve and prevent progression of LVH/LVMI in pediatric patients with CKD 5. The LVH may partially regress after the long-term control of arterial hypertension and severe anemia, as well as after kidney transplantation [29, 34].

In our study, we observed a significantly higher incidence of LVH in the SH group compared to the EU group. LVH is an adaptive response to increased cardiac workload, classically driven by a combination of hypertension and hypervolemia, leading to cardiomyocyte hypertrophy and an expansion of the intercellular matrix. In patients with CKD 5, LVH carries negative prognostic significance, serving as an independent risk factor for arrhythmias, mortality, heart failure, and ischemic heart disease [35].

Probably for constitutional reasons, each method appeared to be inaccurate and unreliable in predicting fluid status in single cases. In fact, LUS, BIS, and IVC-CI failed to predict the correct dry weight in most of the assessments in 3 (13%), 3 (13%), and 6 (26%) patients, respectively. Consequently, LUS and BIS should not be adopted separately from clinical evaluation and other methods to assess a subclinical hypervolemia.

These findings suggest that a three-method approach may be preferable to a two-method approach for the quantification of subclinical hypervolemia. In this pilot study, the multiparametric assessment combining LUS, BIS, and IVC-CI with clinical evaluation appeared to provide a more reliable characterization of fluid status and risk stratification than each method applied individually [36]. Overall, this integrative approach enables the evaluation of hypervolemia across different compartments—LUS reflecting lung congestion, IVC-CI effective circulating volume, and BIS total body extracellular fluid volume—although the results should be interpreted cautiously due to the limited sample size.

### Limitations

(1) It is a monocentric *pilot study* with a small sample size. However, it represents one of the larger studies with contemporary clinical, biochemical, echocardiographic, and multiparametric fluid status assessments in children on dialysis. (2) Two different dialysis modalities (hemodialysis and peritoneal dialysis) were included, introducing potential heterogeneity that may have influenced some of the observed associations. (3) Although LVH can be used as a marker of volume overload to monitor the dry weight of the previous months in dialysis patients, it is influenced by additional factors other than hypervolemia that could have been overlooked. (4) Multiparametric evaluation in patients on peritoneal dialysis was performed during the outpatient visit, about 5–6 h after the end of the previous dialysis session, and not at the peak of interdialytic hypervolemia, immediately before the next one. (5) The time of enrollment in the study did not coincide with the start of dialysis nor with the achievement of a stable dry weight, resulting in some patients already showing LVH at the beginning of the study. (6) We could not perform speckle tracking analysis by echocardiography, which represents an early predictor of myocardial dysfunction in the absence of systolic dysfunction in children with CKD 5 and kidney transplant. Furthermore, regional strain and strain rate have the great advantage to be preload independent, providing precious information on cardiac function irrespective of volume variations [37]. (7) Detailed information on antihypertensive medication use and standardized 24-h ABPM measurements was not collected within the study protocol and could not be retrospectively retrieved; as such, the absence of these data represents an additional limitation of our analysis. (8) Both LUS and IVC-CI are operator-dependent techniques, and inter-observer variability may have influenced some measurements. Although all assessments were performed by trained physicians, this inherent subjectivity represents a further limitation of the study. (9) Blood volume monitoring (crit-line) on hemodialysis machines and serum NT-proBNP levels were not tested in our population and were not included in the proposed definition of subclinical hypervolemia, but they are very useful easy-to-measure and observer-independent methods to detect hypervolemia and should be considered in future studies.

## Conclusions

In conclusion, this pilot study suggests that the three methods—LUS, BIS, and IVC-CI—can be reliably combined to quantify subclinical hypervolemia, showing significant correlations with weight-derived OH and with each other even in patients without overt overload. We propose defining subclinical hypervolemia according to a multiparametric assessment showing at least two out of three abnormal parameters (≥ 5 B-lines at LUS, OH/ECW > 7% at BIS, and/or IVC-CI < 40%) in the absence of clinical signs or symptoms of fluid overload.

Chronic subclinical hypervolemia was associated with a higher frequency of LVH, supporting the potential clinical relevance of this condition. Combining two or more instrumental techniques may help minimize the limitations of single methods and improve the accuracy of fluid status evaluation. These preliminary findings highlight the value of a multimethod approach for detecting and monitoring subclinical hypervolemia in children on dialysis, while emphasizing the need for larger, multicenter studies to confirm its impact on LVH progression and cardiovascular outcomes.

## Supplementary Information

Below is the link to the electronic supplementary material.

Graphical abstract (PPTX 594 KB)

Supplementary file 1 (PNG 39.3 KB)

## Data Availability

The data sets generated during and/or analyzed during the current study are available from the corresponding authors on reasonable request. It will contain deidentified participant data.
