# Supplementary material for: Potential implications of subclinical hypervolemia, identified by a multiparametric approach, in causing left ventricular hypertrophy in clinically euvolemic children on dialysis: a prospective longitudinal pilot study
Source: Pediatr Nephrol. 2026 Jan 30;41(7):2119–29. doi: 10.1007/s00467-026-07159-z (PMC13197374; doi:10.1007/s00467-026-07159-z)
Supplement: Supplementary file 1 — Graphical abstract (PPTX 594 KB) [file 467_2026_7159_MOESM1_ESM.pptx]

## Slide 1
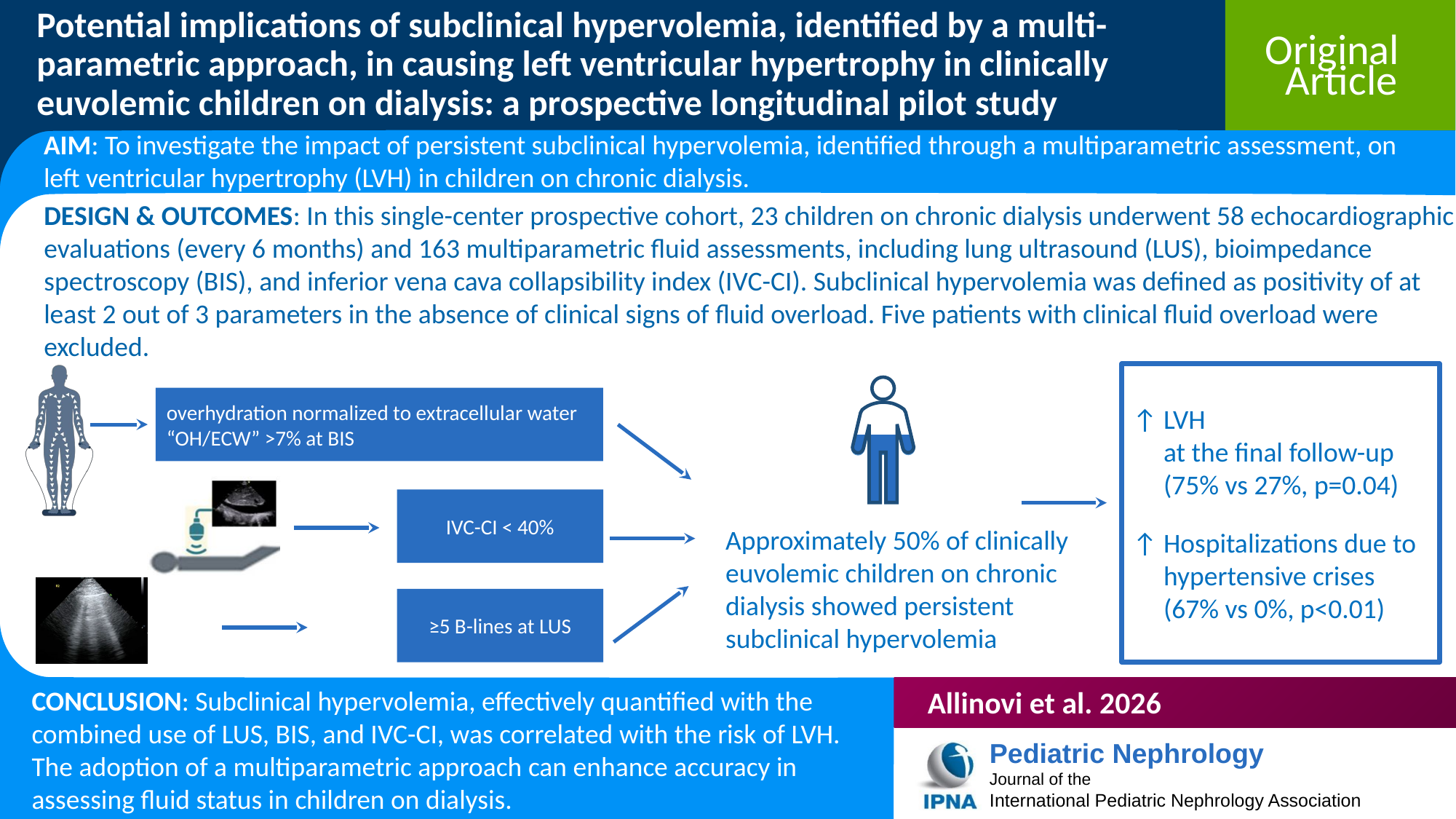

Potential implications of subclinical hypervolemia, identified by a multi-parametric approach, in causing left ventricular hypertrophy in clinically euvolemic children on dialysis: a prospective longitudinal pilot study
AIM: To investigate the impact of persistent subclinical hypervolemia, identified through a multiparametric assessment, on left ventricular hypertrophy (LVH) in children on chronic dialysis.
DESIGN & OUTCOMES: In this single-center prospective cohort, 23 children on chronic dialysis underwent 58 echocardiographic evaluations (every 6 months) and 163 multiparametric fluid assessments, including lung ultrasound (LUS), bioimpedance spectroscopy (BIS), and inferior vena cava collapsibility index (IVC-CI). Subclinical hypervolemia was defined as positivity of at least 2 out of 3 parameters in the absence of clinical signs of fluid overload. Five patients with clinical fluid overload were excluded.
↑ LVH
 at the final follow-up
 (75% vs 27%, p=0.04)
↑ Hospitalizations due to
 hypertensive crises
 (67% vs 0%, p<0.01)
overhydration normalized to extracellular water “OH/ECW” >7% at BIS
IVC-CI < 40%
Approximately 50% of clinically euvolemic children on chronic dialysis showed persistent subclinical hypervolemia
≥5 B-lines at LUS
CONCLUSION: Subclinical hypervolemia, effectively quantified with the combined use of LUS, BIS, and IVC-CI, was correlated with the risk of LVH. The adoption of a multiparametric approach can enhance accuracy in assessing fluid status in children on dialysis.
Allinovi et al. 2026
