# Supplementary figures and images for: Potential implications of subclinical hypervolemia, identified by a multiparametric approach, in causing left ventricular hypertrophy in clinically euvolemic children on dialysis: a prospective longitudinal pilot study
Source: Pediatr Nephrol. 2026 Jan 30;41(7):2119–29. doi: 10.1007/s00467-026-07159-z (PMC13197374; doi:10.1007/s00467-026-07159-z)

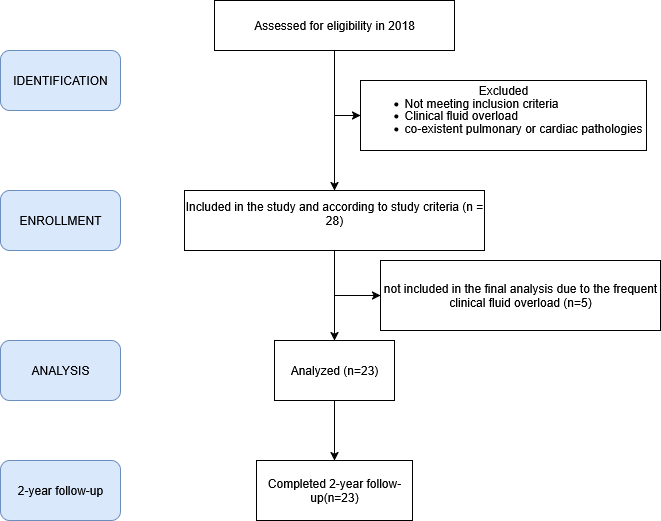

Supplement: Supplementary file 2 — Supplementary file 1 (PNG 39.3 KB) [file 467_2026_7159_MOESM2_ESM.png]
